# Role of Social and App-Related Factors in Behavioral Engagement With mHealth for Improved Well-being Among Chronically Ill Patients: Scenario-Based Survey Study

**DOI:** 10.2196/33772

**Published:** 2022-08-26

**Authors:** Freek Van Baelen, Melissa De Regge, Bart Larivière, Katrien Verleye, Sam Schelfout, Kristof Eeckloo

**Affiliations:** 1 School of Business and Management University College Ghent Ghent Belgium; 2 Strategic Policy Cell Ghent University Hospital Ghent Belgium; 3 Department of Marketing, Innovation and Organisation Faculty of Economics and Business Administration Ghent University Ghent Belgium; 4 Department of Marketing Faculty of Economics and Business Catholic University of Leuven Leuven Belgium; 5 Center for Service Intelligence Ghent University Ghent Belgium; 6 Multidisciplinary Pain Center Ghent University Hospital Ghent Belgium; 7 Department of Public Health and Primary Care Faculty of Medicine and Health Sciences Ghent University Ghent Belgium

**Keywords:** mHealth app, engagement, social influence, app integration, well-being, Belgium, mHealth, behavioral, behavioral engagement, mobile health, mobile health apps, mobile phone

## Abstract

**Background:**

The last decade has seen a considerable increase in the number of mobile health (mHealth) apps in everyday life. These mHealth apps have the potential to significantly improve the well-being of chronically ill patients. However, behavioral engagement with mHealth apps remains low.

**Objective:**

The aim of this study was to describe the behavioral engagement of chronically ill patients with mHealth apps by investigating (1) how it is affected by social factors (ie, physician recommendation) and app-related factors (ie, app integration) and (2) how it affects patient well-being. This study also considers the moderating effect of attachment to traditional health care and the mobile app experience among patients.

**Methods:**

We carried out a scenario-based survey study of chronically ill patients (N=521). A Bayesian structural equation modeling with mediation and moderation analysis was conducted in MPlus.

**Results:**

Both physician recommendations for mHealth app use and app integration have positive effects on the behavioral engagement of chronically ill patients with mHealth apps. Higher behavioral engagement positively affects the hedonic well-being (extent of pleasure) and the eudaemonic well-being (extent of self-efficacy) of chronically ill patients. Mobile app experience, however, positively moderates the relationship between app integration and behavioral engagement, whereas patient attachment to traditional care does not moderate the relationship between physician recommendation and behavioral engagement. Taken together, the proportion of variance explained (R²) equals 21% for behavioral engagement and 52.8% and 62.2% for hedonic and eudaemonic well-being, respectively, thereby providing support for the strong influence of app integration and physician recommendation via the mediation of the patients’ behavioral engagement on both patients’ hedonic and eudaemonic well-being.

**Conclusions:**

Physician recommendation and app integration enable behavioral engagement and promote well-being among chronically ill patients. It is thus important to take social and app-related factors into consideration during and after the development of mHealth apps.

## Introduction

### Background

With the growth in smartphone use and increasing demands from patients for immediate access to web-based services, mobile health (mHealth) apps that allow patients to actively manage their own health through mobile and wireless technologies are on the rise [1-3]. Internationally, the popularity of mHealth apps to support the achievement of health objectives is increasing [4], especially for chronically ill patients [1]. For this group of patients, research suggests that mHealth apps lead to increased confidence in disease management [5,6], improved therapy compliance, better health care outcomes [7], and even reduced costs [6].

Despite the proven impact of mHealth apps on patient well-being [8], patients do not always show high levels of behavioral engagement with them [9-11]. Here, behavioral engagement refers to the adoption and continued usage of mHealth apps by chronically ill adults. User data from popular app stores even show that most mHealth apps are only used a few times before being abandoned [12]. Less than a third of chronically ill patients aged 50 years and older currently use an mHealth app. More than a third of patients have used mHealth apps in the past but have then stopped using them [13]. In an attempt to better understand why behavioral engagement with mHealth apps is low, we explore the drivers of behavioral engagement with mHealth apps.

In line with the technology acceptance model and the unified theory of acceptance and use of technology, several researchers have, in recent years, pointed out the importance of social-related and technology-related factors when explaining behavioral engagement with health care technologies [14,15]. In light of the behavioral engagement with mHealth apps, there has been considerable research on effort and performance expectancy [16]. However, less research has been dedicated to the role of social and app-related drivers of behavioral engagement with mHealth apps. Pham et al [17] also call for more research on the relationship between mHealth engagement and well-being for chronically ill patients.

Against this background, this research characterizes social enablers (ie, physician recommendation) and app-related enablers (ie, app integration) of behavioral engagement with mHealth apps among chronically ill patients, thereby also considering the impact of behavioral engagement with mHealth apps for patient well-being (that is, hedonic well-being defined as the extent of pleasure and eudaemonic well-being defined as the extent of self-efficacy [18]).

### Conceptual Framework and Hypotheses

#### Social Enablers of Patients’ Behavioral Engagement With mHealth Apps

With regard to the social enablers of behavioral engagement with technologies, it is well established that people can affect each other [19]. Specifically, several researchers have shown that behavioral engagement with technologies is—as suggested by the technology acceptance model—a function of social influence [14,20]. Social influence refers to any “change in an individual’s thoughts, feelings, attitudes, or behaviors that results from interaction with another individual or a group” [21]. A key question revolves around which individuals or groups can change an individual’s thoughts, feelings, attitudes, or behaviors in the context of mHealth apps.

Cajita et al [22] have shown that physicians have a significant influence on mHealth app usage among older adults with heart failure. Likewise, Apolinário-Hagen et al [23] demonstrated that physicians significantly affect behavioral engagement with mHealth apps among people with multiple sclerosis. Specifically, patients with different health conditions may interpret the efforts of health care professionals to use mHealth as an incentive to use mHealth themselves [9,24]. Alternatively, patients may show more behavioral engagement with mHealth apps when they receive recommendations for the use of mHealth apps from health care professionals [10], including physicians [25]. As chronically ill patients often have longstanding relationships with their physician and since they tend to follow their physicians’ instructions, we suggest that physician recommendation—that is, the extent to which physicians recommend the use of mHealth apps—can play an important role when engaging these patients with mHealth apps. We hypothesize as follows:

*H1a: Physician recommendation positively affects behavioral engagement with mHealth apps among chronically ill patients.*

As suggested by the diffusion of innovation theory [26], the impact of physician recommendation on behavioral engagement with mHealth apps among chronically ill patients also relates to patients’ own perceptions of the relative advantages of these health care technologies in relation to the idea it supersedes (here, traditional care). If patients are attached to traditional care, the relative advantages of mHealth apps may be less for them. As relative advantage is one of the strongest predictors of the emerging use of technological innovation [27], patients who are more attached to traditional care are less likely to show behavioral engagement with the use of mHealth apps recommended by their physicians. We thus hypothesize as follows:

*H1b: The positive impact of physician recommendation on behavioral engagement with mHealth apps among chronically ill patients will decrease when they are more attached to traditional care.*

#### App-Related Enablers of Patients’ Behavioral Engagement With mHealth Apps

To ensure that health care technologies such as mHealth apps are relevant to patients and health care professionals, several researchers have called for these users to be involved in the app development process [28,29]. In this regard, patients and physicians have emphasized that health care technologies need to enable data exchange with other systems or applications [30-32]. Indeed, health care technologies that lack interoperability (the ability to exchange data with other systems or applications) have been described as information silos [33,34]; the same holds for mobile apps, including mHealth apps, which are not compatible with other systems such as electronic patient records [25,35].

Empirical evidence also suggests that mHealth apps with low levels of interoperability may deteriorate health care outcomes [34,35]. In contrast, allowing mobile apps to exchange data with each other and other digital systems may help to avoid duplication of medical care, increase patient safety, improve the continuity of care, and reduce administrative burdens [33,36]. Moreover, mHealth apps with high levels of interoperability contribute to increased functionality and better experiences for patients [37] while allowing patients to access, store, or make certain information digitally available, thereby making them, to a greater extent, into managers of their own health [31,33,36]. As chronically ill patients often encounter multiple health care providers, we contend that app integration, that is, the extent to which mHealth apps are interoperable is even more important [15,33]. We therefore hypothesize as follows:

*H2a: App integration positively affects behavioral engagement with mHealth apps among chronically ill patients.*

If chronically ill patients have more experience with mobile apps, they are more likely to have tried apps with high levels of interoperability and hence to have experienced how app integration can benefit them. The diffusion of innovation theory [26] confirms that innovations that users can experiment with are (in line with the idea of trialability) more likely to be embraced. Building upon the trialability idea, we contend that the positive effect of app integration on behavioral engagement with mHealth apps is strengthened when patients have more experience with mobile apps. We thus hypothesize as follows:

*H2b: The positive effect of app integration on behavioral engagement with mHealth apps among chronically ill patients increases when such patients have more mobile app experience.*

#### Patient Behavioral Engagement With mHealth Apps for Improved Well-being

The behavioral engagement of patients with health care technologies has been associated with improved well-being [8,38,39]. As widely acknowledged in the well-being literature [18], well-being incorporates hedonic well-being, with its focus on pleasure attainment, and eudaemonic well-being, with its focus on self-realization, that is, the degree to which a person is fully functioning. Research suggests that health care technologies like mHealth apps can contribute to improved hedonic and eudaemonic well-being by providing pleasant experiences to patients and by helping the patients to reach their goals [40,41]. We therefore hypothesize as follows:

*H3a: Behavioral engagement with mHealth apps positively affects the hedonic well-being of chronically ill patients.*

*H3b: Behavioral engagement with mHealth apps positively affects the eudaemonic well-being of chronically ill patients.*

## Methods

### Research Design and Procedure

In this study, we rely upon a scenario-based survey study, which is very common in business research [42] and in technology acceptance studies [43]; an increasing use of scenario-based surveys are also being used in health care [44]. Scenario-based survey studies have the advantage of eliminating the difficulties associated with observation or enactment of events in real life, such as in this study, with undesirable outcomes, and with not reaching a sufficiently large sample size, as can happen when forcing patients to use a nonintegrated app [42]. Compared to recall-based surveys, scenario-based surveys also have the advantage of reducing biases from memory retrieval [45].

This scenario-based survey study involves a between-participant 2×2 design and introduces participants to a scenario. In all scenarios, the patient receives a pamphlet with information on a fictional mHealth app, but the scenarios differed in terms of the recommendation by the physician to use the mHealth app (ie, strong vs weak recommendation to use the mHealth app) and app integration (integrated vs nonintegrated mHealth app). This 2×2 design has 4 different possible scenarios, and each participant was randomly assigned to 1 of these 4 scenarios. After reading the scenario, the participant filled out a questionnaire. The scenarios are detailed in Multimedia Appendix 1.

### Sampling

G*Power 3.1.9 (Heinrich Heine Universität) was used to calculate the required sample size for detecting a medium effect (Cohen *d*=0.5) in an independent sample *t*-test (2-tailed). With 80% power at an α level of .05, a total sample size of 204 participants (51 per group) was needed to test the hypotheses. To achieve the required sample size, respondents with chronic conditions were recruited by more than 60 organizations representing the interests of chronically ill people in the Flemish region of Belgium through sharing the survey in their e-newsletter, website, or Facebook page. Eligible respondents (1) had been diagnosed by their physician with a chronic disease and (2) were aged between 18 and 65 years. These age boundaries were set because the empirical literature identified strong differences in the adoption of technology among young people, adults, and older adults [46]. In total, 722 respondents completed the questionnaire. After quality checks (including age and condition checks and a control question), 521 respondents were retained.

### Ethical Considerations

The Ghent University Hospital review board approved the study protocol (2019/1975-670202042704), and participants were asked for consent.

### Measures

We conducted a web-based survey from March to May 2019. Study data were collected and managed using REDCap electronic data capture tools hosted at Ghent University Hospital [47,48]. The survey involved 5 different constructs, including skip patterns. All constructs were measured using previously validated multi-item scales with proven validity and reliability (See Multimedia Appendix 2 [49-53]). The original scales were translated into Dutch using the forward and backward translation technique. Although validated by previous research, the measurement instrument was further tested to ensure reliability within the study context. Cronbach α values of the validated constructs ranged from .748 to .952 and showed that the reliability requirements were met. Reponses were provided using a 7-point Likert scale, with anchors ranging from 1 (“strongly disagree”) to 7 (“strongly agree”). Finally, the survey included questions about age, gender, and the duration of the chronic condition, as it is common to include these demographics in research relating to chronic conditions [54].

### Analytical Approach

We assessed the experimental interventions by comparing the mean score on a single item measuring physician recommendation (“my physician recommends me to use this app”) and a single item measuring app integration (“this is an integrated app”) between the different scenarios. The mean differences for both interventions were significant. The mean score on a 7-point Likert scale for physician recommendation was 4.24 in the weak physician recommendation scenario versus 5.47 in the strong physician recommendation scenario (*P*=.02). The mean score for app integration was 3.67 in the scenario with a nonintegrated app versus 5.87 in the scenario with an integrated app (*P*=.004).

**Figure 1:**
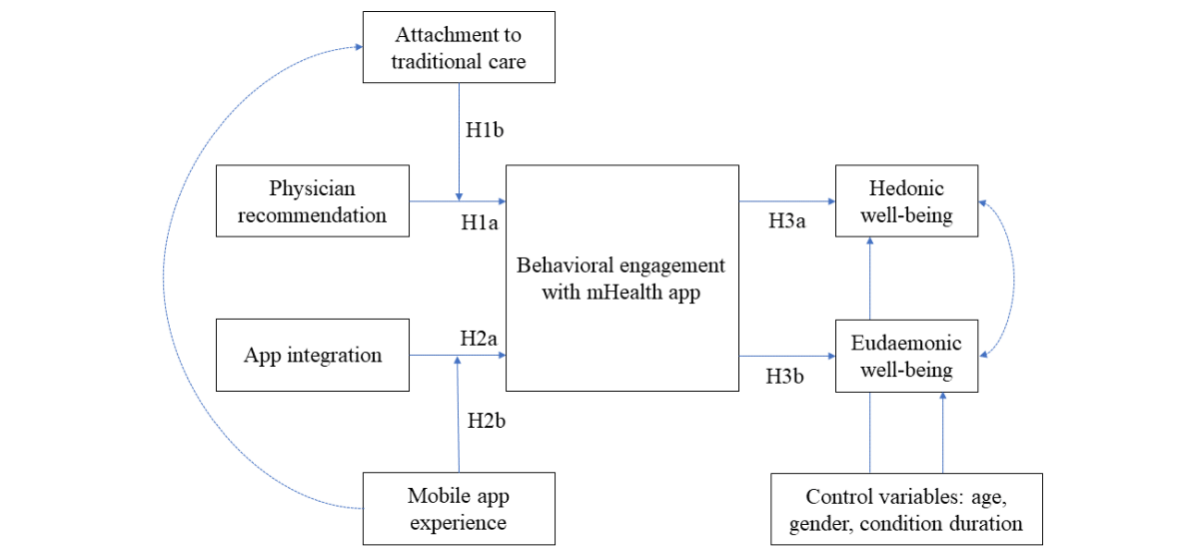
Proposed conceptual model. H: hypothesis; mHealth: mobile health.

To simultaneously test all hypotheses (including drivers, consequences, and moderators), we used a mediation approach [55] with Bayesian estimation [56]. As suggested by Iacobucci [57] and Yuan and MacKinnon [56], the following 3 equations were jointly estimated using structural equation modeling in order to test our proposed conceptual model:



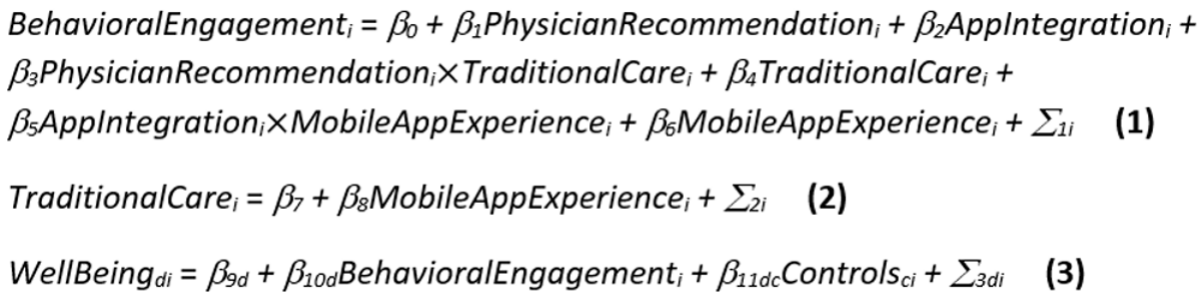



in which the *BehavioralEngagement_i_* denotes the individual *i*’s (*i*=1 to 521) behavioral engagement with mHealth apps and the *WellBeing_di_* denotes the 2 (*d*=1 to 2) well-being dimensions: hedonic well-being (*d*=1) and eudaemonic well-being (*d*=2). β_1_ denotes the effect of the influence of the physician (*PhysicianRecommendation_i_*) on behavioral engagement in order to test H1a, whereas β_2_ denotes the effect of app integration (*AppIntegration_i_*) on behavioral engagement in order to test H2a. β_10d_ *r*epresents the effect of behavioral engagement on both hedonic well-being and eudaemonic well-being and respectively enables testing of H3a and H3b. β_3_ denotes the moderating effect of patient attachment to traditional care on the impact of physician recommendation on patients’ behavioral engagement (*PhysicianRecommendation_i_TraditionalCare_i_*) and allows investigation of H1b. β_5_ denotes the moderating effect of patient mobile app experience on the relationship that the app integration has on the patient’s behavioral engagement (*AppIntegration_i_MobileAppExperience_i_*) and enables investigation of H2b. The *Σ_1i_,* *Σ_2i_,* and *Σ_3di_* are error terms with intercorrelation ρ which, in line with the well-being literature [58], accounts for the interdependency between hedonic and eudaemonic well-being (see Figure 1). *Controls_ci_* is a vector of control variables, including patient age, gender, and duration of condition.

Because of structural equation modeling, the paths as specified in equations 1 to 2 are modeled in combination with the measurement model. The measurement model provided evidence of construct validity and discriminant validity, and additional tests revealed our data to be free from the common method and collinearity biases (See Multimedia Appendix 2 and Multimedia Appendix 3 [55,56,59-67] for more details). In addition, the model convergence was inspected and revealed evidence of a well-fitting model (see Model Convergence Assessment in Multimedia Appendix 3 for more details). Finally, the structural equation models, in line with the technology adoption literature [49], are linked between the mobile app experience and attachment to traditional care, as shown in Figure 1.

## Results

The chronic conditions that were the most prevalent among the respondents were orthopedic and rheumatic diseases (153/521, 29.4%), neurological diseases (133/521, 25.5%), and lung diseases (109/521, 20.9%); 130 respondents (25%) indicated comorbidity. All respondents had a smartphone, but mobile app experience varied; 240 respondents (46.1%) had no mobile app experience, 192 respondents (36.9%) had low-to-moderate mobile app experience, and 89 respondents (17.1%) had high mobile app experience. Table 1 gives an overview of the other demographics of the research participants.

**Figure 2:**
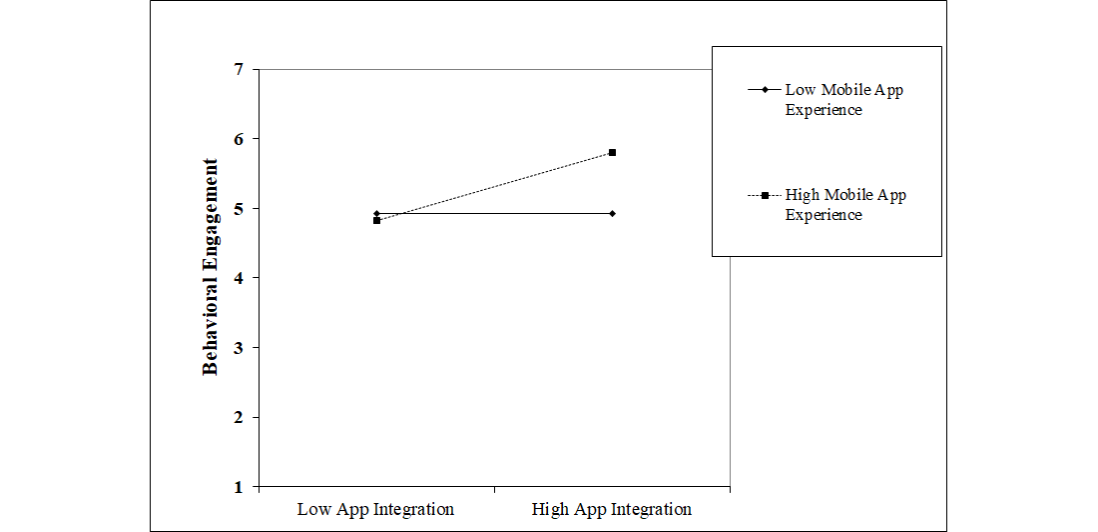
The moderating influence of mobile app experience.

Table 2 presents the model’s findings. The findings of Model 1 reveal that the physician’s recommendation has a positive significant influence on the patient’s behavioral engagement with mHealth apps (β_1_=.325, *P*=.001). H1a is thus supported. The findings also reveal that app integration has a positive effect on behavioral engagement (β_2_=.225, *P*=.02), confirming H2a. As anticipated, behavioral engagement has a positive effect on both hedonic well-being (β_10_1_=.641, *P*=.001) and eudaemonic well-being (β_10_2_=.724, *P*=.001), thereby supporting H3a and H3b. To test the moderating effect of patients’ attachment to traditional care and their mobile app experience, Model 2 in Table 2 reports the parameter estimates of the moderating effects. Model 2 reveals that a patient’s attachment to traditional care only has a direct effect on behavioral engagement (β_4_=*–*.420, *P*=.001) since the interaction terms were found to be insignificant, albeit negative as anticipated (β_3_=–.157, *P*=.14). H1b is thus rejected. With regard to the moderating effect of mobile app experience, our findings show a positive significant moderating effect on the relationship between app integration and behavioral engagement (β_5_=.232, *P*=.02), thereby confirming H2b. Figure 2 depicts this significant relationship, showing that there is a high positive impact of mobile app experience on behavioral engagement with mobile app integration. In addition, our model findings show that gender and condition duration have no significant effects. Interestingly, age is found to only have a negative effect on hedonic well-being (β_11_1_=–.008, *P*=.002), whereas no such effect was observed for eudaemonic well-being. In addition, people with more mobile app experience were found to have less attachment to traditional care (β_8_=–.094, *P*=.01). Finally, the proportion of variance explained (R^2^) equals 21% for behavioral engagement and 52.8% and 62.2% for hedonic and eudaemonic well-being, respectively, thereby providing support for the strong effect of app integration and physician recommendation via mediation of the patients’ behavioral engagement on patients’ hedonic and eudaemonic well-being.

**Table 1:** Participant demographics.

Demographics			All respondents (N=521)		Scenario 1^a^ (n=128)		Scenario 2^b^ (n=123)		Scenario 3^c^ (n=141)		Scenario 4^d^ (n=129)		*P* value	
Age (years), mean (min-max, SD)			44.24 (18-65, 13.11)		45.41 (21-65, 12.47)		44.58 (19-65, 13.46)		43.26 (18-65, 13.21)		43.84 (18-65, 13.32)		.57	
**Gender, n (%)**														.44
	Male	125 (24)		27 (21.1)		34 (27.6)		30 (21.3)		34 (26.4)				
	Female	396 (76)		101 (78.9)		89 (72.4)		111 (78.7)		95 (73.6)				
Condition duration (years), mean (min-max, SD)			12.20 (0-64, 11.09)		11.95 (0-64, 11.95)		14.3 (0-64, 11.65)		11.26 (0-47, 10.83)		11.47 (1-58, 9.90)		.11	

^a^Scenario 1: strong physician recommendation + integrated app.

^b^Scenario 2: weak physician recommendation + nonintegrated app.

^c^Scenario 3: strong physician recommendation + nonintegrated app.

^d^Scenario 4: weak physician recommendation + integrated app.

**Table 2:** Model findings.

		Model 1				Model 2			
		Behavioral engagement	Hedonic well- being	Eudaemonic well-being	Attachment to traditional care		Behavioral engagement	Hedonic well- being	Eudaemonic well-being
**Independent variables, β (*P* value)**									
	Physician recommendation	.325 (.001)^a^	N/A^b^	N/A	N/A		.304 (.002)^a^	N/A	N/A
	App integration	.225 (.02)^a^	N/A	N/A	N/A		.238 (.01)^a^	N/A	N/A
	Behavioral engagement	N/A	.641 (.001)^a^	.724 (.001)^a^	N/A		N/A	.642 (.001)^a^	.723 (.001)^a^
**Control variables, β (*P* value)**									
	Age	N/A	–.008 (.008)^a^	–.004 (.06)	N/A		N/A	–.008 (.002)^a^	–.004 (.06)
	Gender (1=female, 0=male)	N/A	.090 (.16)	–.059 (.24)	N/A		N/A	.089 (.15)	–.070 (.19)
	Condition duration	N/A	.003 (.19)	.004 (.13)	N/A		N/A	.003 (.21)	.003 (.14)
**Testing moderating effects, β (*P* value)**									
	Physician recommendation×Attachment to traditional care	N/A	N/A	N/A	N/A		–.157 (.14)	N/A	N/A
	Attachment to traditional care	N/A	N/A	N/A	N/A		–.420 (.001)^a^	N/A	N/A
	App integration×Mobile app experience	N/A	N/A	N/A	N/A		.232 (.02)^a^	N/A	N/A
	Mobile app experience	N/A	N/A	N/A	–.094 (.01)^a^		.196 (.01)^a^	N/A	N/A
Correlation error term (*P* value)		N/A	0.107 (.001)^a^	0.107 (.001)^a^	N/A		N/A	0.103 (.001)^a^	0.103 (.001)^a^
*R*^2^ (proportion of variance explained; %)		2.8	52.3	61.8	1.4		20.8	52.8	62.2

^a^Effect size (β) is significant.

^b^N/A: not applicable.

## Discussion

### Principal Findings

mHealth has several benefits for patients, especially for those who experience chronic conditions [17]. mHealth can enable patients to manage their condition [68], which is crucial for their well-being [69]. However, beginning to use mHealth apps can be challenging, as many patients have neither experience with nor confidence in using them [17,70]. Although health care technology adoption research has typically focused on the need for user friendliness, usefulness, and performance expectancy [16], we complement this line of research by exploring the importance of social factors (here, physician recommendation) and app-related factors (here, app integration) when behaviorally engaging patients with mHealth and when considering how this engagement impacts their well-being.

With regard to the social factors, we found that physician recommendation positively influences behavioral engagement and consequently, patient well-being. The important role laid out for the physician when behaviorally engaging patients with mHealth apps resonates with evidence about their importance in stimulating other types of patient behavior [71] and enhancing patient well-being [72]. Indeed, the physician is in a unique position to motivate their patients to use mHealth apps, especially when they have longstanding relationships with them [73].

Although we have observed that patients who are more inclined to traditional care are less inclined to use mHealth, it is remarkable to note that these patients can also be motivated to use mHealth apps to the same degree as patients who are more open to receiving modern care (with the use of mobile apps). As such, physicians should be careful not to assume that patients who are more accustomed to traditional care will be harder to motivate to use mHealth. This potential bias should not lead to neglect of this part of the patient population, particularly in the (post-)COVID world where virtual care has become a more integral part of traditional care [74]. Furthermore, although we have focused in this research on the motivating role of the physician, other parties (health care organizations, government, etc) can also be of importance in recommending patients to use mHealth apps. Future research could thus focus on marketing strategies that these parties could deploy to encourage mHealth use. Anderson et al [75] have recently called for an increased use of marketing techniques in health care. Indeed, marketing communication can be instrumental in motivating patients to use new technologies and engage in self-care by creating a positive attitude toward health care technology [76,77].

Besides social factors, our results also demonstrate the importance of app integration. Specifically, integrated apps can have an important positive impact on a patients’ behavioral engagement. The major app stores already offer a staggering 350,000 mHealth apps, most of which remain unsuccessful because of limited behavioral engagement [78]. Designing yet another nonintegrated mHealth app can only add to this pile of underutilized apps. By designing mHealth apps, which have meaningful interaction with existing systems used by the patient (eg, appointment scheduler, patient file, health data), behavioral engagement can be enhanced and the continued usage of an mHealth app can be improved.

In line with previous research [22], this study also shows a moderating role for app experience. Patients with more experience in using apps will place greater value on mHealth apps that are integrated with other health platforms. Given that app experience is rising among all parts of the population [79], the importance of offering integrated apps will only increase in the future. Companies who develop mHealth apps and health care organizations who implement them should focus not only on apps’ appearances and capabilities but also show great care in ensuring that apps are integrated into existing health platforms.

Finally, our data were collected in 2019, shortly before the COVID-19 pandemic. The advantages of mHealth apps during a pandemic have been well-documented [74]. Further, numerous papers on the roles of eHealth, telehealth, and telemedicine in delivering health care services to chronically ill patients during the COVID-19 pandemic have been published [80,81]. The COVID-19 pandemic has shown the importance of mHealth as a means of interacting with patients and providing care. It would be interesting to know how the pandemic has changed the way in which patients use and feel about mHealth apps.

### Limitations and Future Research

Although this study gives clear indications of the importance of physician recommendation and app integration for behavioral engagement and well-being among chronically ill patients, it is not without limitations, and the results should be interpreted accordingly. First, the respondents self-selected to participate in this study. As a result, generalization of the results to a broader population should be done with care. However, because we opted for self-selection, a larger number of participants was recruited. Second, this study uses self-reported data and not actual behavior. This makes the study more vulnerable to self-report biases such as socially desirable answers and information bias. Future research could go beyond scenario-based research and implement the proposed interventions in a randomized controlled trial. Third, this study utilized cross-sectional data, which limits the possibilities of drawing conclusions on causal relationships. Future research could benefit from a longitudinal approach by collecting data at different points in time among the same respondents [12]. It can be envisioned that the engagement of chronically ill patients, in particular, might differ in time, as the severity of their condition or their need for support fluctuates. Fourth, the strength of the relationship between the physician and patient was not included in the study design. Future studies could include measures of this relationship, since the relationship between physician and patient may act as a significant mediator of the relationship between physician recommendation, behavioral engagement with mHealth apps among chronically ill patients, and their well-being.

### Conclusion

An ever increasing number of mHealth apps are being developed by both commercial enterprises and health care organizations. Although these apps can have a positive impact on patient well-being, various studies have shown that simply designing an effective app does not guarantee their adoption by users. This study focused on the importance of physician recommendation and app integration in increasing behavioral engagement and well-being among chronically ill patients. It highlights the importance of app developers considering behavioral engagement during and after the development of mHealth apps. During development, attention should be given to ensuring app integration so that communication and interaction with existing health care systems is possible. Integration is an important characteristic that can encourage patients to start using an app—especially when they are experienced app users. After development, it is important to motivate patients to adopt the mHealth app. This study has shown that physicians have an important role to play in motivating chronically ill patients to engage with mHealth apps.

## Appendix

Multimedia Appendix 1 Presented scenarios.

Multimedia Appendix 2 Table on construct items (wording), factor scores, and composite reliability.

Multimedia Appendix 3 Measurement model, common method bias, collinearity, and robustness tests.

## Abbreviations

mHealth: mobile health
