# Supplementary material for: Role of Social and App-Related Factors in Behavioral Engagement With mHealth for Improved Well-being Among Chronically Ill Patients: Scenario-Based Survey Study
Source: JMIR Mhealth Uhealth. 2022 Aug 26;10(8):e33772. doi: 10.2196/33772 (PMC9463618; doi:10.2196/33772)
Supplement: Multimedia Appendix 1 [file mhealth_v10i8e33772_app1.docx]

Appendix 1: Presented scenarios (translated from Dutch to English)

**SCENARIO 1: STRONG PHYSICIAN RECOMMENDATION - INTEGRATED APPLICATION**

Imagine going to your physician for a check-up of your chronic condition.

During the consultation with your physician, a new app for smartphones comes up for discussion. This app aims to support you in self-management of your chronic condition. In fact, you can use the app to monitor your chronic condition from home.

The app allows you to record essential health information on a daily basis, such as your blood pressure, pulse rate and blood sugar level. The app will automatically record the information and results of your home measurements. In addition, you can also enter your own information that you wish to keep track of in the context of your chronic condition. For example, think about a pain score or exactly what you ate and drank. The same app also integrates your hospital data. As a result, from within the app you can also view your hospital data, consult test results and manage appointments within the hospital. If you give your permission, your physician can follow up on this information electronically.

Your physician gives you a detailed explanation of the app and also gives you a brochure in which you can find all the information. Your physician advises you to read the brochure at home. You then ask for your physician’s opinion of the app. He/she is very positive about the app and strongly recommends that you use it. Finally, your physician explains that using the app is not compulsory.

**SCENARIO 2:** **WEAK PHYSICIAN RECOMMENDATION - STANDALONE APPLICATION**

Imagine going to your physician for a check-up of your chronic condition.

In the waiting room you are looking at a leaflet that refers to a new app for smartphones. This app aims to support you in self-management of your chronic condition. In fact, you can use the app to monitor your chronic condition from home. First of all, the app allows you to record essential health information on a daily basis, such as your blood pressure, pulse rate and blood sugar level. The app will automatically record the information and results of your home measurements. In addition, you can also enter your own information that you wish to keep track of in the context of your chronic condition. For example, think about a pain score or exactly what you ate and drank.

You take the brochure with you to the consultation with your physician. You then ask for your physician’s opinion of the app. He/she indicates that they are indifferent to the app and leaves the choice of whether or not to use it entirely up to you. Finally, your physician explains that using the app is not mandatory.

**SCENARIO 3: STRONG PHYSICIAN RECOMMENDATION - STANDALONE APPLICATION**

Imagine going to your physician for a checkup of your chronic condition.

During the consultation with your physician, a new app for smartphones comes up for discussion. This app aims to support you in self-management of your chronic condition. In fact, you can use the app to monitor your chronic condition from home.

The app allows you to record essential health information on a daily basis, such as your blood pressure, pulse rate and blood sugar level. The app will automatically record the information and results of your home measurements. In addition, you can also enter your own information that you wish to keep track of in the context of your chronic condition. For example, think of a pain score or exactly what you ate and drank.

Your physician explains the app to you in detail and also gives you a brochure in which you can find all the information. Your physician advises you to read the brochure at home. You then ask for your physician’s opinion of the app. He/she is very positive about the app and strongly recommends that you use it. Finally, your physician explains that using the app is not compulsory.

**SCENARIO 4: WEAK PHYSICIAN RECOMMENDATION - INTEGRATED APPLICATION**

Imagine going to your physician to check up on your chronic condition.

In the waiting room you are looking at a leaflet that refers to a new app for smartphones. This app aims to support you in self-management of your chronic condition. In fact, you can use the app to monitor your chronic condition from home.

The app allows you to record essential health information on a daily basis, such as your blood pressure, pulse rate and blood sugar level. The app will automatically record the information and results of your home measurements. In addition, you can also enter your own information that you wish to keep track of in the context of your chronic condition. For example, think about a pain score or exactly what you ate and drank. The same app also integrates your hospital data. As a result, from within the app you can also view your hospital data, consult test results and manage appointments within the hospital. If you give your permission, your physician can follow up on this information electronically.

You take the brochure with you to the consultation with your physician. You then ask for your physician’s opinion of the app. He/she indicates that they are indifferent to the app and leaves the choice of whether or not to use it entirely up to you. Finally, your physician explains that using the app is not mandatory.
