# Supplementary material for: Role of Social and App-Related Factors in Behavioral Engagement With mHealth for Improved Well-being Among Chronically Ill Patients: Scenario-Based Survey Study
Source: JMIR Mhealth Uhealth. 2022 Aug 26;10(8):e33772. doi: 10.2196/33772 (PMC9463618; doi:10.2196/33772)
Supplement: Multimedia Appendix 2 [file mhealth_v10i8e33772_app2.docx]

**APPENDIX 2: Table: Construct items (wording), factor scores (CFA) and Composite Reliabilities (CR)**

Below you can find the factor loadings from CFA and the CR

Behavioral Engagement CR = 0.94 [49]

I intent to use this app in the future 0.915

I will always try to use this app in my daily life 0.912

I plan to use this app regularly 0.937

Eudaimonic Well-Being CR = 0.92 [50,51]

I believe that using the app increases the quality of my life 0.851

I believe that using the app makes my life easier 0.849

I believe that using the app makes me more effective in life 0.857

I believe that using the app helps me to monitor my health status 0.718

I believe that this app is useful in my life 0.890

Hedonic Well-Being CR = 0.94 [52]

This app makes me feel bad-good 0.871

This app makes me feel unpleasant-pleasant 0.886

This app makes me feel disadvantageous-advantageous 0.906

This app makes me feel unfavorable-favorable 0.927

Patient’s attachment to traditional care CR = 0.72 [49]

Traditional care (without apps) has become a habit of mine 0.601

I am attached to traditional care (without apps) 0.804

I need to receive traditional care (without apps) 0.633

Mobile App Experience CR = 0.86 [53]

I have little experience with using apps (reversed) 0.557

I often use apps 0.924

I use many apps 0.952
