# Supplementary material for: Role of Social and App-Related Factors in Behavioral Engagement With mHealth for Improved Well-being Among Chronically Ill Patients: Scenario-Based Survey Study
Source: JMIR Mhealth Uhealth. 2022 Aug 26;10(8):e33772. doi: 10.2196/33772 (PMC9463618; doi:10.2196/33772)
Supplement: Multimedia Appendix 3 [file mhealth_v10i8e33772_app3.docx]

**APPENDIX 3: Measurement Model, Common Method Bias, Collinearity and Model Convergence Assessments**

**Measurement Model.** Exploratory factor analysis was carried out using IBM SPSS Statistics for Windows, version 26 (IBM Corp., Armonk, N.Y., USA). R [59] was used to conduct confirmatory factor analysis. The measurement model for the sample performed well. Specifically, the comparative fit index (CFI), 0.959, and Tucker-Lewis index (TLI), 0.939, were both above common benchmarks of 0.900. Furthermore, the standardized root mean square residual (sRMR) was 0.044 of which is below the advised level of 0.050, thereby reflecting a good fit [60,61]. The individual items and item loadings are presented in Appendix 2. The sample showed convergent validity, since all construct reliabilities (CR) were above 0.60 [62] and all average variances extracted (AVE) exceeded the squared correlations between the constructs, thereby indicating discriminant validity [63].

**Common Method Bias**. We assessed common method bias (CMB) by using the Harman’s single-factor test, which makes use of exploratory factor analysis to check whether a single factor emerges or one general factor accounts for the majority of the covariance among the measures. The results showed four factors, by which the first factor accounted for 45.62% of the variance and all factors together explained 74.57% of the variance, thereby indicating that CMB was not a serious threat to our analyses [64].

**Collinearity Tests**. Multicollinearity was assessed by means of variance inflation factors (VIF) and some robustness tests. First, we ran ordinary least square (OLS) regressions to generate VIF values. All the VIF values of every variable in all equations were below the suggested cut-off value of 5 (i.e., highest VIF was 1.014 for equation 1, 1.079 for equation 2; see [65], p. 204). Second, we performed robustness checks by running different models. Specifically, we ran three models to show that we obtain similar findings for the focal effects (e.g., [66]). Model 1 (M1) and model 2 (M2) are simpler models than model 3 (M3): M1 only included the control variables and tested the impact of the physician’s influence and app integration on behavioral engagement as well as the impact of behavioral engagement on the well-being dependents, M2 added the moderating influence of both patient’s attachment to traditional care and their mobile app experience, and M3 added the direct effect of both the physician’s influence and app integration on the well-being dependents in order to test for partial versus full mediation [55]. M1 and M2 are reported in this paper, and M3 is available upon request. The results across all models with respect to the investigated parameter estimates remained very similar (in terms of signs, significance level, and effect sizes), thereby indicating the robustness of our parameter estimates.

**Model Convergence Assessment**. Bayesian Structural Equation Modeling (SEM) was modeled in MPlus 8.6. In line with Gelman and Rubin [67], we ran three independent MCMC chains with different starting points and 20,000 iterations each, by which the first half is considered as the “burn-in” phase and the remaining half is used to estimate the posterior distribution for the parameters. To assess the convergence of the MCMC algorithm, we inspected the Gelman-Rubin convergence statistic R, autocorrelation plots, and trace plots of the residual variance for the parameter estimates. Specifically, given the last 30,000 iterations (used to estimate the parameters), the largest value of the Gelman-Rubin convergence statistic R ranged between 1.005 and 1.039 (note that Yuan and MacKinnon [56] have suggested that a value of R close to 1 [the highest cut-off being 1.2] is an indication of reasonable convergence). Hence, this investigation provided evidence of the MCMC algorithm’s convergence.
